# Cost-effectiveness analysis of the diarrhea alleviation through zinc and oral rehydration therapy (DAZT) program in rural Gujarat India: an application of the net-benefit regression framework

**DOI:** 10.1186/s12962-017-0070-y

**Published:** 2017-06-08

**Authors:** Samuel D. Shillcutt, Amnesty E. LeFevre, Christa L. Fischer-Walker, Sunita Taneja, Robert E. Black, Sarmila Mazumder

**Affiliations:** 1 Department of International Health, Johns Hopkins Bloomberg School of Public Health, 615 N. Wolfe Street, Baltimore, MD 21205 USA; 2 Centre for Health Research and Development, Society for Applied Studies, 45 KaluSarai, New Delhi, 110016 India

**Keywords:** Diarrhea, Cost-effectiveness, Net-benefit regression, Zinc, Oral rehydration salts, India, Developing countries, Implementation science

## Abstract

**Background:**

This study evaluates the cost-effectiveness of the DAZT program for scaling up treatment of acute child diarrhea in Gujarat India using a net-benefit regression framework.

**Methods:**

Costs were calculated from societal and caregivers’ perspectives and effectiveness was assessed in terms of coverage of zinc and both zinc and Oral Rehydration Salt. Regression models were tested in simple linear regression, with a specified set of covariates, and with a specified set of covariates and interaction terms using linear regression with endogenous treatment effects was used as the reference case.

**Results:**

The DAZT program was cost-effective with over 95% certainty above $5.50 and $7.50 per appropriately treated child in the unadjusted and adjusted models respectively, with specifications including interaction terms being cost-effective with 85–97% certainty.

**Discussion:**

Findings from this study should be combined with other evidence when considering decisions to scale up programs such as the DAZT program to promote the use of ORS and zinc to treat child diarrhea.

**Electronic supplementary material:**

The online version of this article (doi:10.1186/s12962-017-0070-y) contains supplementary material, which is available to authorized users.

## Background

Cost effectiveness analysis (CEA) is useful for evaluating productive efficiency in the health sector [1]. Applications of CEA in countries with strong health technology assessment programs often follow a general continuum from modeling studies to evaluations alongside clinical trials, which can inform revisions to make models more comprehensive [2]. Less attention has been paid to CEA applied to quasi-experimental study designs.

Methods have been developed which combine econometric and economic evaluation techniques by transforming incremental cost-effectiveness ratios into net-benefit statistics for use in regression analysis [3]. Net-benefit regression allows analysts to control for variables that may confound cost-effectiveness, particularly for non-identical groups, handle censored data, calculate confidence intervals parametrically, and to evaluate cost-effectiveness according to subgroups. This method contrasts with more commonly used methods for estimating CEA, which assume that costs and consequences across population groups, geographic areas, and other factors of interest are homogenous for individual study arms or phases.

The growing number of programs being implemented at scale without a contemporaneous comparison arm represents a trend in evaluation efforts from demonstration of efficacy and effectiveness to delivery of interventions integrated within existing health systems. This trend increases the relevance of cost-effectiveness analyses of before and after studies [4–9]; however, these studies are not well established in the literature. The opportunity exists to expand methods for conducting CEAs alongside programs at scale [10] to include approaches which adjust for heterogeneity across study phases and populations and provide a more unbiased measure of cost-effectiveness.

To contribute to the growing body of evidence on the cost-effectiveness of treating child diarrhea with zinc and oral rehydration salts (ORS) in low- and middle-income countries (LMICs), we conducted a cost effectiveness analysis of the diarrhea alleviation through zinc and oral rehydration Therapy (DAZT) program in Gujarat India using net-benefit regression. The objective of the DAZT program was to scale-up coverage of these interventions, and reduce the irrational prescription of more expensive antibiotics and antidiarrheal medicines. Zinc and ORS were delivered through the public and private sectors through a wide variety of channels to maximize market impact. Program evaluation took a before and after study design across 2010–2013, which allowed for the evaluation of zinc itself as an intervention, as well as evaluation of the health systems delivery strategy.

## Study hypothesis and rationale

This study complimented findings from another analysis which explored the economic costs to caregivers of diarrhea management [11]. The hypothesis of the present study was that the DAZT program was cost-effective in delivering zinc and ORS to treat child diarrhea episodes relative to status quo conditions in rural Gujarat existing before the program was initiated when very few children with diarrhea were given zinc. This comparator was chosen to represent the decision about scaling up the program to other districts faced by decision makers. Data suggested that the coverage of zinc and ORS to treat diarrhea among children increased in the intervention districts [12], in addition to lowering the economic burden borne by caregivers across the span of the program [11]. Consistent with other interventions delivered at the community level [13, 14], we expected that program costs were distributed thinly enough across beneficiaries of the program to make economic costs per person good value for money when considered alongside health benefits.

## Methods

### Study setting

The DAZT program introduced zinc and promoted ORS in Gujarat, India for treatment of child diarrhea through both the public and private sectors, utilizing a variety of community and facility based strategies (Additional file 1: Table S1) [12, 15]. The study area consisted of six districts in northeastern Gujarat (Banas Kantha, Dohad, Panch Mahals, Patan, Sabar Kantha, and Surendranagar, population 13 million [16]) and was chosen for its low coverage of zinc, availability of non-governmental organization partners, and priorities set by the Indian Government.

### Program evaluation

Evaluation of the DAZT program consisted of household surveys spanning 2 years to represent the program at maturity, comparing 4200–5080 caregivers and their 2–59 month old children between the start and finish of the study. These surveys assessed only each household’s youngest child, and the latest episode per child occurring in the last 2 weeks. Data from 613 surveys with complete data on length of diarrhea episode were sufficient to perform cost-effectiveness analysis in terms of increases in coverage.

### Power calculation

The main trial was powered to detect differences in coverage of ORS across trial phases [17–19]. For our NBR analysis, power was computed to detect meaningful differences in between a ceiling ratio (λ), or a policy maker’s valuation of a correctly treated episode, and an Incremental Cost-Effectiveness Ratio (ICER) [20], translated to incremental net-benefit (Box1). Participants included in this analysis were restricted to those individuals for whom data on date of recovery were available to allow for consideration of disease duration and recovery during analyses. Power was calculated based on Glick et al. [21] to account for different sample sizes across study phases, and different standard deviations in costs and effects across phases. Alternative hypotheses were tested against a null hypothesis of no difference of cost-effectiveness from λ.

Values for incremental cost and its standard deviations for each study phase were taken from a cluster randomized controlled trial on zinc conducted in Haryana India [22]. Values for incremental coverage were taken from the expected level of scale up of ORS coverage from current levels in the study area. The expected correlation between incremental cost and effect was assumed to be 0.2 based on convention for sociological studies (Ahmed S, personal communication), given an absence of covariance data [21]. The standard Z score for 95% confidence was used for a two-tailed alpha (1.96), with sensitivity analyses on 99, 90, and 80% confidence. Sensitivity analyses were conducted on effectiveness, cost, ceiling ratios (varied between $0.50 and $13.50), and correlation coefficient. Given the available sample size, power calculations indicate that the study had sufficient power to detect a significant difference in incremental cost-effectiveness from λ across major parameterizations including half, twice, and no incremental costs; half, twice, and no incremental effects; correlation coefficients between effects and costs from 0 to 0.7, Z statistics for type 1 and 2 error, and maximum valuations for the treatment of an episode of diarrhea.

#### Box 1

Formulas to calculate power with different sample sizes and different standard deviations for costs and effects in each phase (based on Glick et al. [21, 23])

Formula 1: $$ \Delta {\text{NMB}}\;{ = }\;\Delta {\text{Q}} \times {\text{W}} - \Delta {\text{C}} $$
Formula 2: $$ {\text{r }} = {\text{ n2}}/{\text{n1}} $$
Formula 3: $$ Z_{\beta } = \sqrt {\frac{{rn_{1} \Delta NMB^{2} }}{{(1 + r)[(sd_{c0}^{2} + sd_{c1}^{2} ) + (W^{2} (sd_{q0}^{2} + sd_{q1}^{2} )) - (2W\rho \sqrt {(sd_{c0}^{2} + sd_{c1}^{2} )} \sqrt {sd_{q0}^{2} + sd_{q1}^{2} } }}} - Z_{\alpha /2} $$

**Table:** Variables

ΔC	$6.03	Expected point estimate in the difference in mean cost
ΔQ	0.53	Expected point estimate in the difference in mean effect
ρ	0.2	Correlation in the difference in cost and effect
sd c1	$0.85	Expected standard deviation in the cost in intervention ‘after’ phase
sd c0	$4.17	Expected standard deviation in the cost in control ‘before’ phase
sd q1	$28.53	Expected standard deviation for the effect in intervention ‘after’ phase
sd q0	$36.15	Expected standard deviation for the effect in control ‘before’ phase
W	$0.50–$13.50	Maximum valuation of treatment for an episode of diarrhea
z alpha	1.96	z statistic for the type 1 error
z beta	Result	z statistic for the type 2 error
n1	326	Sample size in the starting point survey
n2	287 (coverage)	Sample size in the endpoint phase

### Costs and effects

Economic costs were assessed from the societal perspective, consisting of those to the caregiver and program costs borne by the non-governmental organizations (NGOs) and the government. Program costs were calculated according to Saving Newborn Lives (SNL) costing guidelines for capital and recurrent costs [24], with data drawn from program records. Costs to caregivers were assessed according to caregiver report through the household surveys, and included direct medical costs, direct non-medical costs (transportation), and indirect costs (wages lost) [25]. Cost-effectiveness according to a caregiver’s perspective was assessed in sensitivity analysis.

Program costs were combined for public sector and private sector activities across starting point, midpoint, and endpoint surveys; and were divided evenly across the caregivers interviewed at the endpoint survey (Table 1). Allocation of program costs across caregivers was scaled to the population of children in the study area (n = 1,188,634), length of the recall period of the survey (cost/child/2 week period). Costs to caregivers were derived from an accompanying study, and declined from $4.04 to $2.49 per child with diarrhea during the program [11]. Program costs components included capital costs (start-up and sustainability, furniture and equipment, and training), and recurrent costs (personnel, vehicles, buildings, zinc and supplies). Annualized capital costs were considered for the number of years that they were in use—2 years for starting point and midpoint costs, and 1 year for endpoint costs. Recurrent costs were considered for starting point, midpoint, and endpoint phases. Salary costs for service providers were derived per year from the provider assessment survey (Table 2 shows these in more detail).

**Table 1:** Program and provider costs associated with the DAZT program by year

Program costs	Year 1	Year 2
Startup		
Private sector startup capital	$6116	$6116
Private sector startup recurrent	$170,221	
Public sector startup capital	$82,873	$82,873
Public sector startup recurrent	$247,798	
Implementation phase		
Private sector year 1 recurrent	$506,966	
Private sector year 2 recurrent		$479,374
Public sector year 1 capital	$52,764	$52,764
Public sector year 1 recurrent	$295,842	
Public sector year 2 capital		$22,967
Public sector year 2 recurrent		$512,747
Salary costs year 1	$1,068,126	
Salary costs year 2		$1,068,126
Total costs	$2,424,589	$2,224,965
Total		$4,649,554
Proportion of population under 5		9%
Number of children under 5		1,188,634
2 weeks		0.038461538
Total cost per person		$0.150

**Table 2:** Public sector personnel costs attributed to the DAZT program by year

	Facility based providers		Community based providers	
	Medical officers (MO)	Auxiliary nurse midwife (ANM)	Accredited social health activist (ASHA)	Anganwadi worker (AWW)
Mean annual salary	$9,760.46	$4593.56	$277.51	$1030.60
Total working time per week (h)	48	45	3	5
% Time on diarrhea	2%	1%	11%	9%
% Time on zinc	0.26%	0.04%	1.98%	1.59%
Total providers	4237	16,949	42,373	42,373
Total cost	$108,794	$31,842	$232,821	$694,670
Grand total				$1,068,126

Capital components were annualized using factors from the WHO-CHOICE study discounted at 3% as the rate for a risk-free investment [26] and recommendations for economic evaluation in LMICs [27]. In the absence of other data, items were assumed to last the duration of the program. Currency was inflated to 2014 base year United States dollars using consumer price indices from the International Monetary Fund (IMF), and converted using mid-year exchange rates from [28].

In the absence of data on mortality and long-term sequelae from diarrhea, outcomes were assessed in terms of episodes correctly treated with zinc and ORS. Episodes were assessed according to caregiver report of those beginning or resolving within the 2 weeks prior to survey.

#### Cost-effectiveness according to net-benefit regression

All children included in the baseline and endline surveys of the study were included. For each child, the net-benefit statistic was calculated by multiplying the variable representing whether a child with diarrhea was treated with zinc (E_i_) by the monetary valuation of an increase in coverage by one individual with diarrhea (λ), then subtracting the economic costs associated with that individual (C_i_).

1 $$ E\left( {y = NMB_{i} } \right) = \lambda \times E_{i} - C_{i} $$

The net-benefit statistic was used as the dependent variable in a series of simple and multivariable linear regressions. In these formulations, the coefficient on the treatment variable (δ) represented incremental net-benefit of the program relative to the initial survey. Simple linear regression on δ alone, representing study phase, was conducted to show that the incremental net-benefit is the same when calculated in this approach as when calculated in the standard deterministic approach.

2 $$ E\left( {y = NMB_{i} } \right) = \alpha + \delta t_{i} + \varepsilon_{i} $$

Multiple linear regression was then conducted to evaluate net-benefit when controlling for covariates (x_ij_).

3 $$ E\left( {y = NMB_{i} } \right) = \alpha + \sum\limits_{j = 1}^{p} {\beta_{j} x_{ij} \,+\, } \delta t_{i} + \varepsilon_{i} $$

Finally, incremental net-benefit for subgroups was calculated by interacting each parameter with the treatment variable (γ).

4 $$ E\left( {y = NMB_{i} } \right) = \alpha + \sum\limits_{j = 1}^{p} {\beta_{j} x_{ij} \,+\, } \delta t_{i} + t_{i} \sum\limits_{j = 1}^{p} {\gamma_{j} x_{ij} \,+\, } \varepsilon_{i} $$

Different values of λ were tested in sensitivity analysis, including the extreme case of 0 to test negative costs as the response variable. An effects-only regression to represent the scenario where λ equals infinity was not conducted as the outcome is binary and logistic regression coefficients would not be comparable to other model specifications.

#### Cost-effectiveness acceptability curves

Cost effectiveness acceptability curves (CEACs) were constructed to depict the probability that the DAZT intervention was cost-effective according to different levels of λ according to established methods [3].

### Regression modeling

Models were tested according to assumptions of linear regression to inform the appropriate econometric approach. Despite skewness apparent in the residual in quantile–quantile plots, ordinary least squares (OLS) estimation was deemed appropriate given that it produces unbiased estimates due to asymptotic normality. Breusch Pagan/Cook Weisberg tests indicated that the residuals did not have constant variance at different points along the regression line, and visual inspection of residual versus fitted plots confirmed heteroskedasticity. Therefore robust standard errors were used to ensure accurate measurement of p-values. Variance inflation factors indicated that there was no collinearity between covariates.

The importance of highly influential points, meaning those with the greatest potential for altering the slope of the regression line, was checked by calculating DFBETAs for adjusted models with interaction terms. Individuals with the top 10 DFBETA scores were excluded in sensitivity analysis, as using the 2/√n threshold excluded well over 10% of the data. Comparison of AIC/BIC scores indicated that omitting these points slightly improved model fit, although no difference was seen in the significance of the coefficient on the treatment variable. Since these extreme cases were considered important to the analysis to make results comparable to the CEA according to bootstrapping, reference case calculations were performed on the full dataset of children with diarrhea and known date of recovery.

**Fig. 1:**
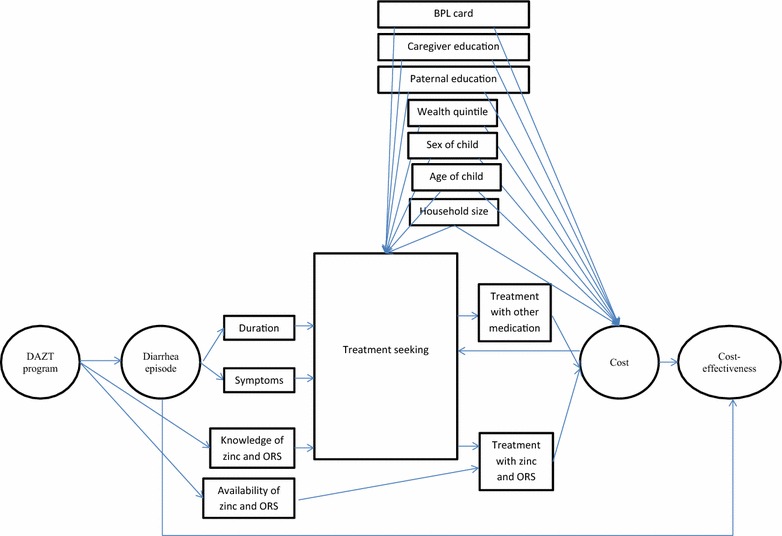
Conceptual framework

Covariates for the model were chosen according to those collected in the study that were relevant for an adapted form of the Andersen and Newman conceptual framework for treatment seeking [29], with hypotheses about the direction of each variable shown in Additional file 2: Table S2. Three main models were specified; (1) using a linear regression with endogenous treatment effects approach based on a conceptual framework (Fig. 1—developed from [10], with hypotheses tied to the literature in Additional file 2: Table S2), (2), using the full set of covariates, and (3) using the set of covariates used in a related study [30]. The approach using linear regression with endogenous treatment effects was used as the reference case, which used probit regression in the reduced form equation and linear regression in the structural equation. Rationale for this choice was that treatment seeking from private providers was expected to be endogenous with a bidirectional relationship with costs, and statistical evidence using a Heckman test confirmed this expectation at λ = $0 [31]. Having blood in the stool was chosen as the instrumental variable since it was correlated with seeking care from a private provider, and was not expected to be correlated with the error term of the structural equation. Sensitivity analyses were performed testing education as a continuous variable, and collapsing treatment seeking into a single dichotomous variable. Specification using automatic selection procedures was ruled out since extensive arguments exist against both ‘significance testing approaches’ [32–35] and ‘change in estimate approaches’ [33, 36, 37]. In addition, automatic selection approaches including bivariable regressions and stepwise backwards selection revealed different sets of explanatory variables for the model at different levels of λ making this approach meaningless in the absence of a consensus value for λ.

To construct a wealth index for assessing the effect of assets on net-benefit, principal components analysis was performed according to standard methods [38, 39], using the component with the highest eigenvalue to construct a scale for classifying caregivers into quintiles. Descriptive statistics and regression analyses were calculated in Stata 13, with program costing and acceptability curves calculated in Microsoft Excel.

## Results

### Descriptive statistics and program costs

Survey participants were 18 and 21 months old at the starting point and endpoint surveys respectively, and had diarrhea for a mean of 3 days at the time of interview. Initially, less than 1% of children received appropriate diarrhea treatment, and only 22% of their caregivers had more than primary education. Among the 39% of caregivers that sought care outside of the home, 78% went to private providers (Table 3).

**Table 3:** Descriptive statistics of the sample of children under 5 with diarrhea from six districts of Gujarat: continuous variables

Variable	Baseline (N = 287)	Endline (N = 326)	Statistical tests	
	Mean	Mean	F test	p value
Child age (months)	18.01	21.43	9.920	0.002
Household size	6.826	6.623	0.790	0.377
Duration of diarrhea	3.24	3.14	0.430	0.514
Coverage with zinc	1.47%	11.42%	27.048	0.000
Coverage with ORS	8.65%	20.88%	30.010	0.000
Coverage with zinc and ORS	0.33%	9.46%	52.432	0.000
Female child	22.02%	23.65%	0.411	0.522
Paternal primary education	38.34%	47.15%	4.046	0.046
Paternal secondary education	18.60%	26.26%	3.447	0.065
Mother primary education	21.70%	32.79%	10.409	0.002
Mother secondary education	6.85%	10.93%	2.764	0.098
Scheduled caste	5.38%	9.95%	4.140	0.044
Scheduled tribe	17.29%	16.48%	0.938	0.334
Other backwards caste	17.78%	22.02%	0.392	0.532
Knowledge about ORS	25.12%	37.36%	16.444	0.000
Knowledge about zinc	2.94%	10.28%	19.606	0.000
Below poverty line card	20.39%	25.94%	1.109	0.294
Poorest wealth quintile	15.33%	7.50%	16.814	0.000
Very poor wealth quintile	9.46%	11.42%	0.121	0.728
Poor wealth quintile	8.97%	10.93%	0.174	0.678
Less poor wealth quintile	6.20%	14.19%	13.408	0.000
Least poor wealth quintile	6.69%	8.97%	0.640	0.425
Blood in stool	3.26%	3.43%	0.067	0.796
Public facility provider	6.85%	8.81%	0.286	0.5935
Public community based provider	1.79%	7.01%	14.033	0.000
Private provider	30.18%	32.95%	0.310	0.578

** Significant at p < 0.05, * Marginally significant at p < 0.10

Program costs totaled $3.56 per child in recurrent costs, and $0.26 per child in annualized capital costs. Capital costs were higher in the public sector, which was responsible for block, district, and state level training workshops to launch the program, and for the initial seed supply of ORS and zinc. The largest component of costs to the private sector were for subcontracts to other NGOs and a pharmaceutical company for training pharmaceutical representatives in zinc use and the short messaging service (SMS) system to monitor sales, and managing of ‘DAZT corners’.

Sample statistics for costs and health outcomes are presented in Table 4. Cost per average person in the sample decreased by $0.12, and diarrhea episodes correctly treated with zinc and ORS increased by 17% leading to a tradeoff between higher costs and greater effectiveness.

**Table 4:** Sample statistics from deterministic economic evaluation

Group variable	Mean	SD	SE
Overall analysis			
Initial survey (N = 287)			
Cost	$3.72	$8.52	$0.50
Effect	0.70%	8.33%	0.49%
Correlation	0.005		
Endline (N = 326)			
Cost	$3.60	$4.61	$0.26
Effect	17.79%	38.30%	2.12%
Correlation	0.029		
Incremental differences between phases			
Cost difference	−$0.12		
Effect difference	17.09%		

### Regression outputs

**Fig. 2:**
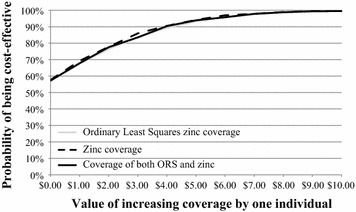
Unadjusted cost-effectiveness acceptability curves

**Fig. 3:**
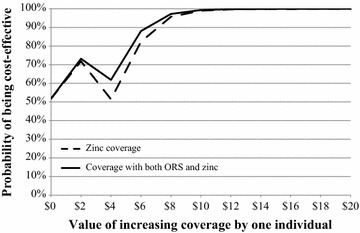
Adjusted cost-effectiveness acceptability curves using a linear regression with endogenous treatment effects approach to defining covariates

**Fig. 4:**
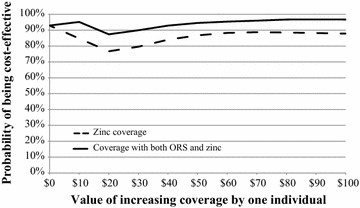
Adjusted cost-effectiveness acceptability curves with interaction terms using a linear regression with endogenous treatment effects approach to defining covariates

The program was cost-effective above λ > $5.50 with 95% certainty for all simple linear specifications tested (Fig. 2; Table 5). Adjusting the model for the set of covariates retained in the linear regression with endogenous treatment effects approach revealed a threshold of $7.50 per correctly treated episode above which the program was cost-effective with 95% certainty (Fig. 3; Table 6), and $10 for the specification with the full set of covariates (Additional file 3: Table S3). When interaction terms were added to the model using the linear regression with endogenous treatment effects approach, the program was cost-effective with above 85% certainty across levels of λ (Fig. 4; Table 7). These results were robust to sensitivity analysis using the caregiver’s perspective (Additional file 3: Table S3). Since seeking treatment from a private provider was endogenous, the linear regression with endogenous treatment effects model with interaction terms should be considered the reference case and least prone to bias. These results were variable in other approaches to choosing and defining independent variables, although many of the acceptability curves were meaningless. Including education as a continuous variable and categorizing treatment seeking as a single dichotomous variable had little effect on results (Additional file 4: Figure S1, Additional file 5: Table S4, Additional file 6: Figure S2, Additional file 7: Figure S3, Additional file 8: Figure S4, Additional file 9: Figure S5, Additional file 10: Figure S6).

**Table 5:** Unadjusted net benefit of the DAZT program relative to (baseline) conditions before the program using ORS and zinc coverage as the effectiveness measure

Variable	NB with λ = $0	NB with λ = $2	NB with λ = $4	NB with λ = $6	NB with λ = $8	NB with λ = $10
Constant term	−3.72 [0.57] (0.00)	−3.70 [0.57] (0.00)	−3.69 [0.54] (0.00)	−3.67 [0.56] (0.00)	−3.66 [0.57] (0.00)	−3.65 [0.57] (0.00)
Study phase	0.12 [0.65] (0.43)	0.46 [0.65] (0.24)	0.80 [0.63] (0.10)	1.15 [0.67] (0.04)	1.49 [0.68] (0.01)	1.83 [0.68] (0.00)
Adjusted R-squared	−0.002	−0.001	0.002	0.005	0.009	0.014
Wald chi-2	0.03	0.51	1.65	2.92	4.76	7.23
Prob > chi-2	0.852	0.474	0.199	0.088	0.029	0.007
AIC	4079	4082	4093	4113	4140	4173
BIC	4088	4090	4102	4121	4149	4182
y-hat-squared	0.825	0.397	0.145	0.041	0.010	0.002
[Se] (p value)

**Table 6:** Adjusted net benefit of the DAZT program relative to (baseline) conditions existing before the program using a linear regression with endogenous treatment effects approach to defining covariates and ORS and zinc coverage as the effectiveness measure

Variable	NB with λ = $0	NB with λ = $4	NB with λ = $8	NB with λ = $12	NB with λ = $16	NB with λ = $20
Constant term	0.94 [0.69] (0.09)	−5.07 [1.03] (0.00)	−5.82 [1.14] (0.00)	−6.68 [1.32] (0.00)	−7.45 [1.50] (0.00)	−8.10 [1.67] (0.00)
Study phase	0.02 [0.54] (0.48)	0.10 [0.35] (0.38)	0.80 [0.42] (0.03)	1.49 [0.51] (0.00)	2.10 [0.60] (0.00)	2.66 [0.67] (0.00)
Household size	0.16 [0.11] (0.07)	0.21 [0.13] (0.05)	0.22 [0.14] (0.06)	0.23 [0.15] (0.06)	0.24 [0.16] (0.07)	0.25 [0.18] (0.08)
Female child	0.39 [0.47] (0.20)	0.45 [0.59] (0.22)	0.43 [0.62] (0.25)	0.41 [0.67] (0.27)	0.38 [0.72] (0.30)	0.36 [0.78] (0.32)
Child age (months)	0.04 [0.02] (0.02)	0.06 [0.03] (0.02)	0.06 [0.03] (0.02)	0.06 [0.03] (0.03)	0.06 [0.03] (0.04)	0.06 [0.03] (0.05)
Paternal primary education	−0.56 [0.73] (0.22)	−1.72 [1.03] (0.05)	−1.98 [1.10] (0.04)	−2.25 [1.20] (0.03)	−2.51 [1.31] (0.03)	−2.75 [1.41] (0.03)
Paternal secondary education	0.86 [0.57] (0.07)	0.89 [0.73] (0.11)	0.86 [0.77] (0.13)	0.84 [0.83] (0.16)	0.81 [0.89] (0.18)	0.79 [0.96] (0.20)
Maternal primary education	0.42 [0.54] (0.22)	0.86 [0.68] (0.10)	0.98 [0.72] (0.09)	1.10 [0.79] (0.08)	1.23 [0.86] (0.08)	1.36 [0.92] (0.07)
Maternal secondary education	−0.68 [0.61] (0.13)	−0.37 [0.90] (0.34)	−0.30 [0.97] (0.38)	−0.22 [1.06] (0.42)	−0.15 [1.16] (0.45)	−0.08 [1.27] (0.48)
BPL card	−0.18 [0.52] (0.37)	−0.77 [0.62] (0.11)	−0.99 [0.65] (0.07)	−1.21 [0.71] (0.04)	−1.42 [0.76] (0.03)	−1.62 [0.82] (0.02)
Wealth index—2nd quintile	−1.01 [0.97] (0.15)	−1.80 [1.14] (0.06)	−1.99 [1.22] (0.05)	−2.20 [1.32] (0.05)	−2.38 [1.42] (0.05)	−2.53 [1.52] (0.05)
Wealth index—3rd quintile	−0.80 [0.54] (0.07)	−0.75 [0.78] (0.17)	−0.89 [0.86] (0.15)	−1.03 [0.96] (0.14)	−1.15 [1.07] (0.14)	−1.27 [1.17] (0.14)
Wealth index—4th quintile	−0.87 [0.55] (0.06)	−0.88 [0.87] (0.15)	−0.82 [0.97] (0.20)	−0.75 [1.10] (0.25)	−0.66 [1.24] (0.30)	−0.54 [1.37] (0.35)
Wealth index—5th quintile	−0.91 [0.99] (0.18)	−1.37 [1.26] (0.14)	−1.18 [1.35] (0.19)	−1.00 [1.47] (0.25)	−0.80 [1.60] (0.31)	−0.58 [1.73] (0.37)
Seek treatment from a public facility	−1.26 [0.51] (0.01)	2.95 [0.82] (0.00)	3.83 [0.95] (0.00)	4.79 [1.12] (0.00)	5.71 [1.31] (0.00)	6.56 [1.49] (0.00)
Seek treatment from a public community based provider	0.02 [0.67] (0.49)	4.16 [1.17] (0.00)	5.85 [1.33] (0.00)	7.60 [1.55] (0.00)	9.34 [1.78] (0.00)	11.04 [2.02] (0.00)
Seek treatment from a private provider	−5.87 [0.62] (0.00)	4.56 [0.86] (0.00)	5.76 [1.01] (0.00)	7.17 [1.28] (0.00)	8.46 [1.55] (0.00)	9.56 [1.79] (0.00)
Wald chi-2	166.360	71.460	102.980	99.840	90.100	84.700
Prob > chi-2	0.000	0.000	0.000	0.000	0.000	0.000
AIC	4791	4419	4526	4668	4818	4963
BIC	4946	4574	4681	4822	4972	5117
[Standard error] (p value)

**Table 7:** Net benefit of the DAZT program relative to (baseline) conditions existing before the program

Variable	NB with λ = $0	NB with λ = $20	NB with λ = $40	NB with λ = $60	NB with λ = $80	NB with λ = $100
Constant term	0.20 [1.01] (0.42)	−8.42 [1.73] (0.00)	−10.67 [2.28] (0.00)	−10.97 [4.66] (0.01)	−7.26 [10.27] (0.24)	−6.26 [6.52] (0.17)
Study phase	1.53 [1.04] (0.07)	2.50 [2.18] (0.13)	4.92 [3.35] (0.07)	7.97 [4.73] (0.05)	11.02 [5.99] (0.03)	13.51 [7.36] (0.03)
Treatment-covariate interactions						
Household size	0.30 [0.17] (0.04)	0.36 [0.20] (0.03)	0.36 [0.23] (0.06)	0.33 [0.26] (0.10)	0.26 [0.28] (0.17)	0.22 [0.25] (0.19)
Female child	0.33 [0.77] (0.33)	1.06 [1.23] (0.19)	1.20 [1.45] (0.20)	1.21 [1.63] (0.23)	0.91 [1.69] (0.30)	0.83 [1.56] (0.30)
Child age (months)	0.04 [0.03] (0.09)	0.06 [0.05] (0.12)	0.06 [0.06] (0.16)	0.05 [0.06] (0.21)	0.03 [0.06] (0.30)	0.02 [0.05] (0.33)
Paternal primary education	−0.53 [1.20] (0.33)	−2.63 [1.67] (0.06)	−3.68 [2.06] (0.04)	−4.12 [2.64] (0.06)	−3.44 [3.86] (0.19)	−3.40 [2.96] (0.13)
Paternal secondary education	2.03 [1.02] (0.02)	1.42 [1.24] (0.13)	1.11 [1.41] (0.21)	0.93 [1.42] (0.26)	0.98 [1.27] (0.22)	0.88 [1.37] (0.26)
Maternal primary education	1.22 [0.87] (0.08)	2.25 [1.12] (0.02)	2.55 [1.33] (0.03)	2.60 [1.55] (0.05)	2.16 [1.88] (0.12)	2.05 [1.50] (0.09)
Maternal secondary education	−1.42 [1.27] (0.13)	−0.84 [1.70] (0.31)	−0.81 [2.02] (0.34)	−0.99 [2.20] (0.33)	−1.59 [2.18] (0.23)	−1.92 [2.03] (0.17)
BPL card	−0.39 [0.94] (0.34)	0.45 [1.09] (0.34)	0.82 [1.28] (0.26)	1.10 [1.36] (0.21)	1.17 [1.38] (0.20)	1.40 [1.48] (0.17)
Wealth index—2nd quintile	−1.65 [1.60] (0.15)	−2.50 [1.90] (0.09)	−2.68 [2.31] (0.12)	−2.48 [2.66] (0.18)	−1.58 [3.14] (0.31)	−1.08 [2.74] (0.35)
Wealth index—3rd quintile	−0.88 [0.76] (0.12)	−0.04 [1.34] (0.49)	0.12 [1.66] (0.47)	0.12 [1.71] (0.47)	−0.21 [1.52] (0.45)	−0.28 [1.27] (0.41)
Wealth index—4th quintile	−0.61 [0.85] (0.24)	−2.22 [1.57] (0.08)	−2.75 [1.88] (0.07)	−2.78 [1.96] (0.08)	−1.87 [2.40] (0.22)	−1.53 [1.87] (0.21)
Wealth index—5th quintile	−1.70 [1.92] (0.19)	−1.40 [2.53] (0.29)	−0.89 [2.95] (0.38)	−0.22 [3.25] (0.47)	0.73 [3.34] (0.41)	1.53 [3.52] (0.33)
Seek treatment from a public facility	−0.44 [0.74] (0.28)	4.98 [1.27] (0.00)	7.20 [1.97] (0.00)	8.29 [3.43] (0.01)	7.13 [6.52] (0.14)	7.43 [4.81] (0.06)
Seek treatment from a public community based provider	0.25 [1.33] (0.42)	3.83 [1.96] (0.03)	4.84 [2.72] (0.04)	4.91 [3.49] (0.08)	3.11 [5.11] (0.27)	2.52 [3.27] (0.22)
Seek treatment from a private provider	−5.58 [1.00] (0.00)	7.91 [1.00] (0.00)	12.06 [2.35] (0.00)	12.76 [7.20] (0.04)	6.64 [17.30] (0.35)	4.88 [10.79] (0.33)
Covariates						
Household size	−0.30 [0.18] (0.05)	−0.13 [0.34] (0.35)	0.08 [0.51] (0.43)	0.31 [0.69] (0.33)	0.57 [0.88] (0.26)	0.82 [1.08] (0.22)
Female child	0.04 [0.90] (0.48)	−1.57 [1.93] (0.21)	−2.03 [2.58] (0.22)	−2.24 [3.19] (0.24)	−1.96 [3.61] (0.29)	−2.09 [4.17] (0.31)
Child age (months)	−0.02 [0.03] (0.30)	0.00 [0.06] (0.48)	−0.01 [0.08] (0.47)	−0.01 [0.10] (0.45)	−0.02 [0.11] (0.43)	−0.02 [0.13] (0.43)
Paternal primary education	−0.47 [1.26] (0.35)	−0.24 [2.46] (0.46)	−0.23 [3.41] (0.47)	−0.86 [4.34] (0.42)	−2.53 [5.44] (0.32)	−3.73 [6.09] (0.27)
Paternal secondary education	−2.18 [1.13] (0.03)	−0.96 [1.74] (0.29)	−0.72 [2.40] (0.38)	−0.78 [3.07] (0.40)	−1.35 [3.89] (0.36)	−1.53 [4.55] (0.37)
Maternal primary education	−1.32 [0.94] (0.08)	−1.99 [1.83] (0.14)	−1.46 [2.47] (0.28)	−0.57 [3.10] (0.43)	0.99 [3.82] (0.40)	2.06 [4.16] (0.31)
Maternal secondary education	1.44 [1.43] (0.16)	1.01 [2.63] (0.35)	1.29 [3.65] (0.36)	1.83 [4.58] (0.35)	2.84 [5.37] (0.30)	3.53 [6.24] (0.29)
BPL card	0.51 [1.03] (0.31)	−4.10 [1.70] (0.01)	−6.30 [2.39] (0.00)	−7.94 [3.18] (0.01)	−8.58 [4.26] (0.02)	−10.10 [4.40] (0.01)
Wealth index—2nd quintile	1.64 [1.58] (0.15)	0.91 [2.31] (0.35)	0.77 [3.38] (0.41)	0.41 [4.39] (0.46)	−0.30 [5.47] (0.48)	−0.99 [6.61] (0.44)
Wealth index—3rd quintile	0.32 [1.02] (0.38)	−1.86 [2.55] (0.23)	−2.68 [3.45] (0.22)	−3.36 [4.17] (0.21)	−3.63 [4.73] (0.22)	−4.29 [5.56] (0.22)
Wealth index—4th quintile	−0.10 [1.14] (0.46)	3.50 [2.62] (0.09)	5.97 [3.58] (0.05)	7.76 [4.50] (0.04)	8.38 [5.69] (0.07)	9.69 [6.35] (0.06)
Wealth index—5th quintile	1.63 [2.08] (0.22)	1.31 [3.41] (0.35)	2.96 [4.65] (0.26)	4.88 [5.81] (0.20)	7.28 [6.98] (0.15)	9.10 [8.46] (0.14)
Seek treatment from a public facility	−1.30 [1.09] (0.12)	3.54 [2.56] (0.08)	6.00 [3.95] (0.06)	8.06 [5.35] (0.07)	9.90 [6.40] (0.06)	12.41 [7.61] (0.05)
Seek treatment from a public community based provider	−0.57 [1.56] (0.36)	9.61 [3.23] (0.00)	18.71 [4.83] (0.00)	27.72 [6.10] (0.00)	36.94 [6.85] (0.00)	46.38 [8.21] (0.00)
Seek treatment from a private provider	−0.60 [0.94] (0.26)	4.07 [2.90] (0.08)	3.12 [3.04] (0.15)	1.56 [4.01] (0.35)	0.20 [3.79] (0.48)	0.19 [4.19] (0.48)
Wald chi-2	195.5	289.7	168.35	112.8	134.02	140.12
Prob > chi-2	0.000	0.000	0.000	0.000	0.000	0.000
AIC	4824	4976	5566	5984	6302	6558
BIC	5107	5259	5849	6267	6585	6841
[standard error] (p value)						

Multivariable regression with interaction terms using a linear regression with endogenous treatment effects approach define covariates and ORS and zinc coverage as the effectiveness measure

## Discussion

Net-benefit regression provides a useful framework for evaluating the cost-effectiveness of the DAZT program while adjusting for confounding variables (Additional file 11: Table S5). Reference case results indicate that the program was cost-effective with 85% certainty relative to conditions at the initial survey across levels of λ. Claxton has argued that 51% certainty about cost-effectiveness is sufficient to maximize net-benefit to society [40], although decision makers may be risk averse about making an incorrect decision, in which case a threshold closer to 95% would be more appropriate. Expected Value of Perfect Information (EVPI) analysis could be used to evaluate the level of uncertainty that should be acceptable to decision makers.

Subgroup effects were observed, but should not be emphasized as hypotheses about subgroups were not specified before this analysis [41], and data dredging is never recommended [42]. In addition, zinc is inexpensive, microbes do not develop resistance to zinc [43], and there are often costs associated with rationing medicine [44]; reducing the risks of, and supporting the case for widespread use. It is unlikely that policy will be made to allocate zinc according to sociodemographic variables [45], and treating sociodemographic groups differently can lead to problems with human rights and discrimination [42]. For these reasons, we did not emphasize results from sub-group analysis, despite this being a methodological functionality of net-benefit regression.

Currently, there is a lack of cost-effectiveness analyses evaluating interventions on PubMed in the Indian context indicating key gaps in the evidence base. Therefore results from this study are likely to be considered in an ad-hoc fashion by program managers and policy makers instead of being used in a more structured sector-wide decision making process [47]. In addition, this analysis is limited to cost-effectiveness, not cost-utility analysis, making it more useful to the diarrhea community than more general decision makers in the health sector. As the evidence base of economic evaluation strengthens in India, results may be useful for its newly formed health technology assessment organization [48], pharmaceutical pricing negotiations, regulation of insurance, providing bargaining power to public health centers and procurement agencies, promotion of India’s generic drug industry, and education and research purposes among other things [49]. A database of pharmacoeconomic studies has been proposed for India, in which this study could be included [49].

The proliferation of large scale public health programs in the developing world evaluated without control groups provides increasing opportunities to conduct net-benefit regression when undertaking economic evaluation. In addition, this methodology is useful for randomized trials that fail to produce a balanced allocation of covariates. However, the application of net-benefit regression in a LMIC context is relatively new, particularly with regard to maternal, newborn and child health programs. Hounton and colleagues have conducted two studies [50, 51] to evaluate public health interventions in a LMIC context, including programs for skilled attendance at childbirth and community based insurance in Burkina Faso.

Consistent with WHO recommendations [52], zinc has been found to be cost-effective for treatment of non-severe child diarrhea based on modeled evidence from Tanzania [53], and an evaluation of a social franchise platform from Myanmar [54]. Findings from hospital based efficacy trials have demonstrated mixed results; indicating a trend towards costs savings and cost-effectiveness while failing to achieve statistical significance [56, 57] and in a further trial not achieving cost-effectiveness [55]. However, these studies have been small in scale and in two instances zinc was provided with copper [55, 56]. Our study contributes to the evidence base about the use of zinc in real world sector-wide conditions, suggesting that may be cost-effective for scaling up coverage depending on how much decision makers value scaling up coverage of zinc and ORS by one appropriately treated patient. These findings are consistent with previous evidence in favor of the cost-effectiveness of close to client treatment of child diarrhea with zinc and ORS in a community setting [53, 54]. However, scaling up requires a variety of supply and demand side factors for effectiveness to be achieved in other areas such as manufacturing capacity, supply chains, absorptive capacity, and measures to stimulate care seeking and reduce demand for antibiotics [58–60]. Decisions will also be affected by contextual factors beyond diarrhea considerations.

Evidence on the cost-effectiveness of zinc for child diarrhea is particularly relevant in the Indian context where the government has set policy for a national program to provide zinc to all affected children over 3 months old [61], and diarrhea was the third leading killer of children under 5 years old nationwide in 2010 [62]. With diarrhea falling from the second to fourth largest killer of children worldwide in 2 years [63, 64], this study adds to needed evidence to expedite progress in addressing this solvable problem.

### Limitations

As data were not collected describing the date that diarrhea resolved past the administration of the survey, and imputation was not done to estimate these values, the analysis was conducted on only 53% of diarrhea episodes in the sample. It was not possible to determine whether the 47% of episodes for which complete duration was not assessed were acute or persistent, although only one case in the subset evaluated in complete case analysis could be classified as persistent. However, adequate power existed to detect a meaningful difference in cost-effectiveness in complete case analysis, and limiting the analysis to the acute cases clearly defined by the survey was deemed appropriate as it is consistent with other analyses of this program [65].

The evidence base on which we designed the conceptual framework was limited as net-benefit regression has not been done previously on this research question. As a result, rationale for each variable included was based on literature evaluating treatment seeking or cost of treatments. In addition, rationale for hypotheses for each variable was developed after the specifications for the dissertation draft of this model were estimated. Other variables that have been found to be associated with these outcomes but that were not measured in our survey included breastfeeding [66], perceived severity of the diarrhea episode [67, 68], distance to health provider [69, 70], birth order [70, 71], antenatal care of the mother from trained providers [71], expected cost according to perceived diarrhea status [66], timing of careseeking [66], and frequency of episodes [66]. Further research may be conducted to determine the association of these covariates with net-benefit and their influence on incremental results. Specifications were not defined fully in advance of estimating models. While preliminary results did not affect hypotheses we formulated based on literature, the fact that we did not prespecify them or anticipate some of the challenges to specification is not ideal when estimating econometric models, recognizing that both statistical theory and improving model fit are considered in applied econometrics [72]. After analysis on our study was completed, new guidelines were released, which advocate the use of a societal perspective and health systems perspective as the reference case [73] and could be estimated from our data. During this project, methods were identified that could be used to extrapolate deaths averted from coverage levels to which our results could be compared [10]. Other endogeneity tests exist, and further work should be done on a similar dataset that was collected in Uttar Pradesh during the course of this program.

This study’s uncontrolled before and after study design limits measurement of impact. Imbalances between before and after phases were controlled for through the regression approach; however, no mathematical adjustment can control for unobserved characteristics in uncontrolled study designs. In the case of this study, secular trends may have influenced results such as patterns of zinc availability that would have occurred in the absence of the program due to the national recommendation. To some extent these effects may have been countered by secular trends in costs, which may have been expected to increase given current trends of economic development in Gujarat. Therefore, we did not present analyses using episodes averted as the effectiveness measure, despite having sufficient power to do so. Authors can provide results showing that the program was cost-effective using this denominator on request. To protect against contamination to the extent possible, the program was monitored for the influence of similar program activities in DAZT areas. However, the Hawthorne effect may have magnified effects relative to the impact of the program without evaluation activities taking place. To some extent these effects may have been countered by secular trends in costs, which may have been expected to increase given current trends of economic development in Gujarat. Given that the Government of India approved zinc for use in pediatric diarrhea in 2006, evidence from cluster randomized trials suggested both effectiveness and cost-effectiveness [22, 46], and the objective of this study was to evaluate zinc when delivered at scale, our study design was appropriate for these conditions.

The fact that this analysis evaluates cost-effectiveness analysis defined in natural units limits capacity to compare results to cost-utility analyses of other interventions. However, outcomes measured in terms of natural units are often used in net-benefit regression [3, 50, 51], and outputs of this analysis are important in other ways. The finding that controlling for confounders and sets of those included had an effect on cost-effectiveness, with different model specifications producing heterogeneous results, challenges the robustness of conclusions from calculations performed according to non-stratified non-regression based methods.

## Conclusions

This study evaluated the cost-effectiveness of the DAZT program to treat child diarrhea in Gujarat India. The main finding from this study was that the program was cost-effective with 95% certainty above $5.50 and $7.50 per appropriately treated child in the unadjusted and adjusted models respectively, with specifications including interaction terms being cost-effective with 85–97% certainty. Further work is needed to evaluate if programs such as DAZT are cost-effective at scale in improving population health, which provide cost-utility estimates for comparison to other interventions.

## Additional files

**Additional file 1: Table S1.** DAZT intervention components according to activity in Gujarat.

**Additional file 2: Table S2.** Hypotheses about variables.

**Additional file 3: Table S3.** Adjusted net benefit of the DAZT program relative to (baseline) conditions existing before the program using the full set of covariates and ORS and zinc coverage as the effectiveness measure 1.

**Additional file 4: Figure S1.** Adjusted cost-effectiveness acceptability curves using the full set of covariates.

**Additional file 5: Table S4.** Net benefit of the DAZT program relative to (baseline) conditions existing before the program—multivariable regression with interaction terms with full set of covariates and with ORS and zinc coverage as the effectiveness measure.

**Additional file 6: Figure S2.** Adjusted cost-effectiveness acceptability curves with interaction terms using the full set of covariates.

**Additional file 7: Figure S3.** Adjusted cost-effectiveness acceptability curves with interaction terms using an instrumental variable approach to defining covariates from a caregiver’s perspective.

**Additional file 8: Figure S4.** Adjusted cost-effectiveness acceptability curves with interaction terms using the full set of covariates from the caregiver’s perspective.

**Additional file 9: Figure S5.** Cost-effectiveness acceptability curves: Rheingans selection of variables [30].

**Additional file 10: Figure S6.** Cost-effectiveness acceptability curves: Rheingans selection of variables adjusted with interaction terms.

**Additional file 11: Table S5.** Net benefit of the DAZT program relative to status quo—multivariable regression with interaction terms with Rheingans et al. [30] set of interaction terms: ORS and zinc coverage as effectiveness measure.

## Abbreviations

AIC: Akaike’s information criterion
ASHA: accredited social health activist
AWW: Anganwadi worker
BIC: Bayesian information criterion
CEA: cost-effectiveness analysis
CEAC: cost-effectiveness acceptability curves
DAZT: diarrhea alleviation through zinc and ORS therapy program
ICER: incremental cost-effectiveness ratio
IMF: International Monetary Fund
LMIC: low and middle income country
NGO: non-Governmental Organization
OLS: ordinary least squares
ORS: oral rehydration salts
SMS: short messaging service
SNL: saving newborn lives
UNICEF: United Nations Children’s Fund
WHO: World Health Organization
WHO-CHOICE: World Health Organization CHOosing Interventions that are Cost-Effective
