# Supplementary material for: Cost-effectiveness analysis of the diarrhea alleviation through zinc and oral rehydration therapy (DAZT) program in rural Gujarat India: an application of the net-benefit regression framework
Source: Cost Eff Resour Alloc. 2017 Jun 8;15:9. doi: 10.1186/s12962-017-0070-y (PMC5465559; doi:10.1186/s12962-017-0070-y)
Supplement: Supplementary file 1 — Additional file 1: Table S1. DAZT intervention components according to activity in Gujarat. [file 12962_2017_70_MOESM1_ESM.docx]

**Web Table 1.** DAZT intervention components according to activity in Gujarat

| **Sectors** | **Program activities** |
| --- | --- |
| **Public sector** | **Micronutrient Initiative (MI)** |
| State-level  policy changes | • Permission to implement DAZT was formalized through Memorandums of Cooperation between MI and the state government, and MI and the Department of Health and Family Welfare.  • Commitment from the Department of Women and Child Development  • Added zinc to NRHM guidelines and essential drug list |
| Programmatic  planning | • National Rural Health Mission (NRHM) Program Implementation Plans (PIPs) were changed to include the procurement of zinc and ORS |
| Training | • Three levels of training were conducted including (1) district level supervisors, (2) Block level supervisors and health workers, and (3) ASHAs and AWWs. Trios, a Delhi-based agency, conducted training. |
| Supply | • Supply was provided by two pharmaceutical companies including Healthy Life Pharma and FDC limited assuming that the public sector would treat 10%-15% of diarrhea cases.  • Kits contained two ORS sachets and 14 taste masked zinc tablets, a measuring cup, and an informational leaflet for caregivers. |
| Procurement | • Healthy Life Pharma and FDC limited provided the first procurement of kits.  • In phase 1 (2011) MI provided a tender scheme to procure ORS and zinc, and in phase 2 (2012) MI limited its provision to zinc only (government procured ORS).  • In 2013, the state governments disbursed funds to all districts to purchase zinc.  • ANMs may have used supply procured from sources other than MI. |
| Incentives | • Incentives were delivered to ASHAs, AWWs, and ANMs at monthly meetings to increase attendance rates |
| Distribution | • Supplies were distributed from Healthy Life Pharma to district medical stores, to district hospitals or block offices/CHC/PHC, to HSC-ANMs and CDPOs, to ASHAs and AWWs  • ANMs informed PHC block level supervisors about needs; supplies were redistributed from areas of surplus to areas of shortage |
| Monitoring and  supervision | • Supportive supervisors and MI divisional coordinators provided supportive supervision at the district, block, sub-center, and village levels in the form of data validation and capacity building  • These mechanisms complemented existing monitoring mechanisms of the public health system  • Supervisors attended monthly meetings of ASHAs, AWWs, ANMs, spent at least 18 days monitoring field staff visits, provided staff with hands on training when necessary, analyzed service provider knowledge and skills, stock status, and caregiver compliance with treatment |
| **Private sector** | **Family Health International-360 (FHI-360)** |
| Policy changes | • Memorandums of understanding were signed with prominent professional medical organizations (IAP, IMA, and other local medical associations).  • Partnered with NGOs, pharmaceutical companies, and homeopathic and alternative medicine associations |
| Programmatic  planning | An implementation plan was developed which involved a push and pull strategy— push: changed prescription among key opinion leaders in the medical community and created IEC materials with medical experts about diarrhea management and marketed ORS and zinc to RMPs and drug sellers. pull: natural demand creation for ORS and zinc within this group |
| Training | • NGO and pharmaceutical staff trained for three days in diarrhea epidemiology, importance of zinc and ORS, correct dosage and regulatory guidelines, and promotional strategies for effective product placement  • Professional organization were provided with continuing medical education  • DAZT corner staff were trained on selected topics from the three day training schedule |
| Supply | • Local manufacturers were linked with informal providers in designated areas |
| Procurement | • RMPs procured zinc from West Coast Pharmaceuticals and generic brands from NGOs, with procurement plans accounting for different levels of demand according to season |
| Incentives | • Pharma companies provided field representatives with commissions of 2 Rupees for each sale above 200 |
| Distribution | • Generic distributors supplied District Coordinator offices, which distributed to the Tehsil Coordinator based on demand |
| DAZT corners | • Staffed informational booths in private clinics and hospitals to create awareness among caregivers and remind providers to prescribe zinc |
| Monitoring and  supervision | • Monthly NGO and pharma staff meetings, validation of data and reports, SMS messaging from the field  • FHI staff attended monthly meetings, district coordinators spent a lot of time in the field working with new staff |
| **UNICEF** |  |
| Advocacy | • Partnered with the Indian Academy of Pediatrics (IAP) Gujarat chapter to endorse zinc and ORS  • Collaborated with IAP to publish their endorsement of zinc in their bulletin, which reaches 800 pediatricians in Gujarat and thousands of RMPs  • With the Indian Medical Association (IMA), held a state level advocacy workshop, two district level workshops on childhood diarrhea prevention and treatment for promoting ORS and zinc. Participation of major stakeholders - state health officials, leading pediatricians, local media, and DAZT partner.  • Collaborated with IMA to publish their endorsement in October 2012 Bulletin of the IMA, along with FAQs provided by UNICEF  • Influenced government to scale up zinc and ORS statewide in their annual Program Implementation Plan 2013-2014 |
| Training | • Demonstrated use of zinc for diarrhea management in six districts  • Incorporate zinc into fast tracked IMNCI training of front line health workers  • Sensitization workshop for field staff and NGO partners, who sensitized Rural Medical Practitioners (RMPs) and Indigenous Systems of Medical Practitioners (ISMPs) to use zinc and ORS,  • Nine continuing medical education (CME) workshops with private practitioners for the use of zinc and ORS (2 with pediatricians, 7 with RMPs) |
| Supply | • Established mechanisms to make supply available to NGO partners  • Monitored availability of ORS and zinc at Village Health and Nutrition Days (VHND)  • Supported capacity building of 13 medical colleges to monitor VHNDs |
| Additional support | • Development of a frequently asked questions booklet  • Provide information from 33 districts and 8 corporations on the availability of zinc and ORS among front line health workers  • Supported diarrhea prevention activities through Water Sanitation and Hygiene (WASH) programs including Communication for Development (C4D) and Child Development and Nutrition (CDN). These programs worked through different line ministries. |
