# Supplementary material for: Cost-effectiveness analysis of the diarrhea alleviation through zinc and oral rehydration therapy (DAZT) program in rural Gujarat India: an application of the net-benefit regression framework
Source: Cost Eff Resour Alloc. 2017 Jun 8;15:9. doi: 10.1186/s12962-017-0070-y (PMC5465559; doi:10.1186/s12962-017-0070-y)
Supplement: Supplementary file 2 — Additional file 2: Table S2. Hypotheses about variables. [file 12962_2017_70_MOESM2_ESM.docx]

**Web Table 2.** Hypotheses about variables

| Variables | | Direction of coefficient | | Outcome measured in literature | | Source | |
| --- | --- | --- | --- | --- | --- | --- | --- |
| **Predisposing factors** | |  | |  | |  | |
| **Demographic characteristics** | | | |  | |  | |
| Larger household size | | + | | Spending | | [[2](#_ENREF_2)] | |
| Female child | | - | | Care seeking from public providers Delay in care seeking Willingness to pay | | [[3](#_ENREF_3)] [[4](#_ENREF_4), [5](#_ENREF_5)] [[6](#_ENREF_6)] | |
| Child age | | - | | Care seeking  Level of health facility care seeking  Direct costs | | [[3](#_ENREF_3)] [[7](#_ENREF_7)] [[8](#_ENREF_8)] [[9](#_ENREF_9)] [[10](#_ENREF_10), [11](#_ENREF_11)] | |
| **Characteristics of the social structure** | | | | | | |  |
| Paternal primary education | - | | | | Care seeking | | [[10](#_ENREF_10), [12](#_ENREF_12)] |
| Paternal secondary education | - | | | | Expect an education gradient | |  |
| Caregiver primary education | - | | | |  | | [[11](#_ENREF_11)] |
| Caregiver secondary  education | - | | | | Higher education associated with seeking care in general | | [[13](#_ENREF_13)] |
| Scheduled caste | not specified | | | | Less likely to seek treatment | | [[14](#_ENREF_14), [15](#_ENREF_15)] |
| Scheduled tribe | not specified | | | |  | |  |
| Other backwards caste | not specified | | | |  | |  |
| **Caregiver knowledge** |  | | | |  | |  |
| Knowledge about ORS | - | | | | Care seeking | | [[4](#_ENREF_4)] |
| Knowledge about zinc | - | | | |  | |  |
| **Enabling factors** |  | | | |  | |  |
| Study phase | - | | | | Episodes <= 4 days, DALYs averted | | [[16](#_ENREF_16)] [[17-19](#_ENREF_17)] |
| Below poverty line card | + | | | | Care seeking | | [[4](#_ENREF_4)] |
| Wealth index - 2nd quintile | not specified | | | | Care seeking | | [[4](#_ENREF_4), [15](#_ENREF_15)] |
| Wealth index - 3rd quintile | not specified | | Cost | | | | [[7](#_ENREF_7)] |
| Wealth index - 4th quintile | not specified | | Institutional delivery | | | | [[20](#_ENREF_20)] |
| Wealth index - 5th quintile | not specified | |  | | | |  |
| **Need factors** |  | |  | | | |  |
| Duration diarrhea <6 days | + | | Care seeking | | | | [[21](#_ENREF_21)] |
| Duration diarrhea ≥6 days | + | |  | | | |  |
| Blood in the stool | + | | Care seeking | | | | [[3](#_ENREF_3)] |
| **Source of care** |  | |  | | | |  |
| Public provider - facility | + | |  | | | |  |
| Public provider - community | + | |  | | | |  |
| Private provider | + | |  | | | |  |
| **Treatment given** |  | |  | | | |  |
| Given ORS | + | |  | | | |  |
| Given zinc | + | | Cost | | | | [[22](#_ENREF_22)] |
