# Supplementary material for: Cost-effectiveness analysis of the diarrhea alleviation through zinc and oral rehydration therapy (DAZT) program in rural Gujarat India: an application of the net-benefit regression framework
Source: Cost Eff Resour Alloc. 2017 Jun 8;15:9. doi: 10.1186/s12962-017-0070-y (PMC5465559; doi:10.1186/s12962-017-0070-y)
Supplement: Supplementary file 3 — Additional file 3: Table S3. Adjusted net benefit of the DAZT program relative to (baseline) conditions existing before the program using the full set of covariates and ORS and zinc coverage as the effectiveness measure 1. [file 12962_2017_70_MOESM3_ESM.docx]

**Web Table 3.** Adjusted net benefit of the DAZT program relative to (baseline) conditions existing before the program using the full set of covariates and ORS and zinc coverage as the effectiveness measure`1

| **Variable** | **NB with  = $0** | **NB with  = $4** | **NB with  = $8** | **NB with  = $12** | **NB with  = $16** | **NB with  = $20** |
| --- | --- | --- | --- | --- | --- | --- |
| Constant term | 2.50 [1.28]  (0.03) | 2.36 [1.24]  (0.03) | 2.22 [1.31]  (0.04) | 2.08 [1.43]  (0.07) | 1.94 [1.52]  (0.10) | 1.80 [1.67]  (0.14) |
|  |  |  |  |  |  |  |
| Study phase | 0.07 [0.60]  (0.46) | 0.46 [0.57]  (0.21) | 0.86 [0.62]  (0.09) | 1.25 [0.65]  (0.03) | 1.64 [0.68]  (0.01) | 2.04 [0.74]  (0.00) |
| Household size | 0.16 [0.11]  (0.07) | 0.18 [0.11]  (0.05) | 0.20 [0.12]  (0.05) | 0.22 [0.13]  (0.04) | 0.24 [0.14]  (0.04) | 0.26 [0.15]  (0.04) |
| Female child | 0.42 [0.45]  (0.17) | 0.41 [0.45]  (0.18) | 0.39 [0.46]  (0.20) | 0.37 [0.50]  (0.23) | 0.35 [0.51]  (0.25) | 0.33 [0.53]  (0.27) |
| Child age (months) | 0.04 [0.02]  (0.01) | 0.03 [0.02]  (0.01) | 0.03 [0.02]  (0.02) | 0.03 [0.02]  (0.04) | 0.03 [0.02]  (0.06) | 0.03 [0.02]  (0.08) |
| Paternal primary education | -0.70 [0.78]  (0.19) | -0.78 [0.77]  (0.16) | -0.86 [0.79]  (0.14) | -0.95 [0.84]  (0.13) | -1.03 [0.86]  (0.12) | -1.11 [0.95]  (0.12) |
| Paternal secondary education | 0.86 [0.56]  (0.06) | 0.81 [0.53]  (0.06) | 0.75 [0.55]  (0.09) | 0.69 [0.62]  (0.13) | 0.63 [0.63]  (0.16) | 0.58 [0.70]  (0.21) |
| Maternal primary education | 0.42 [0.53]  (0.21) | 0.53 [0.55]  (0.17) | 0.63 [0.59]  (0.14) | 0.74 [0.61]  (0.11) | 0.84 [0.65]  (0.10) | 0.94 [0.68]  (0.08) |
| Maternal secondary education | -0.96 [0.69]  (0.08) | -0.89 [0.71]  (0.11) | -0.82 [0.73]  (0.13) | -0.75 [0.76]  (0.16) | -0.68 [0.83]  (0.21) | -0.61 [0.96]  (0.26) |
| Scheduled caste | -0.49 [0.77]  (0.26) | -0.68 [0.74]  (0.18) | -0.87 [0.72]  (0.11) | -1.06 [0.81]  (0.10) | -1.25 [0.89]  (0.08) | -1.45 [1.01]  (0.08) |
| Scheduled tribe | 0.19 [0.97]  (0.42) | 0.08 [0.99]  (0.47) | -0.04 [0.98]  (0.49) | -0.15 [1.08]  (0.44) | -0.26 [1.14]  (0.41) | -0.38 [1.27]  (0.38) |
| Other backwards classes | -0.35 [0.98]  (0.36) | -0.40 [0.90]  (0.33) | -0.46 [0.89]  (0.30) | -0.51 [1.01]  (0.31) | -0.56 [1.06]  (0.30) | -0.62 [1.20]  (0.30) |
| Knowledge about ORS | 0.32 [0.47]  (0.25) | 0.38 [0.48]  (0.22) | 0.43 [0.51]  (0.20) | 0.48 [0.52]  (0.18) | 0.53 [0.54]  (0.16) | 0.59 [0.59]  (0.16) |
| Knowledge about zinc | -1.26 [0.63]  (0.02) | 0.10 [0.61]  (0.43) | 1.47 [0.72]  (0.02) | 2.84 [0.81]  (0.00) | 4.20 [0.99]  (0.00) | 5.57 [1.20]  (0.00) |
| BPL card | -0.33 [0.55]  (0.27) | -0.42 [0.55]  (0.23) | -0.50 [0.58]  (0.19) | -0.59 [0.61]  (0.16) | -0.68 [0.65]  (0.15) | -0.77 [0.67]  (0.13) |
| Wealth index - 2nd quintile | -1.21 [0.94]  (0.10) | -1.24 [0.93]  (0.09) | -1.28 [0.98]  (0.10) | -1.31 [1.03]  (0.10) | -1.34 [1.04]  (0.10) | -1.37 [1.08]  (0.10) |
| Wealth index - 3rd quintile | -0.76 [0.63]  (0.12) | -0.89 [0.66]  (0.09) | -1.02 [0.69]  (0.07) | -1.15 [0.76]  (0.06) | -1.29 [0.81]  (0.06) | -1.42 [0.86]  (0.05) |
| Wealth index - 4th quintile | -0.78 [0.60]  (0.10) | -0.64 [0.63]  (0.15) | -0.51 [0.69]  (0.23) | -0.38 [0.79]  (0.32) | -0.25 [0.85]  (0.39) | -0.11 [0.93]  (0.45) |
| Wealth index - 5th quintile | -1.01 [1.24]  (0.21) | -0.84 [1.25]  (0.25) | -0.67 [1.29]  (0.30) | -0.49 [1.36]  (0.36) | -0.32 [1.40]  (0.41) | -0.15 [1.51]  (0.46) |
| Duration of diarrhea | -0.78 [0.34]  (0.01) | -0.75 [0.34]  (0.01) | -0.71 [0.36]  (0.02) | -0.68 [0.37]  (0.03) | -0.65 [0.35]  (0.03) | -0.62 [0.36]  (0.04) |
| Blood in the stool | -0.93 [0.85]  (0.14) | -0.65 [0.86]  (0.23) | -0.37 [0.91]  (0.34) | -0.10 [0.94]  (0.46) | 0.18 [1.09]  (0.44) | 0.46 [1.23]  (0.36) |
| Seek treatment from a public facility | -0.46 [0.60]  (0.22) | -0.09 [0.60]  (0.44) | 0.29 [0.68]  (0.34) | 0.66 [0.74]  (0.19) | 1.03 [0.87]  (0.12) | 1.40 [0.94]  (0.07) |
| Seek treatment from a public community based provider | 0.99 [0.75]  (0.10) | 1.96 [0.79]  (0.01) | 2.93 [0.96]  (0.00) | 3.91 [1.16]  (0.00) | 4.88 [1.39]  (0.00) | 5.85 [1.58]  (0.00) |
| Seek treatment from a private provider | -4.36 [0.47]  (0.00) | -4.39 [0.51]  (0.00) | -4.41 [0.50]  (0.00) | -4.44 [0.55]  (0.00) | -4.46 [0.58]  (0.00) | -4.49 [0.63]  (0.00) |
|  |  |  |  |  |  |  |
| Adjusted R-squared | 0.148 | 0.153 | 0.170 | 0.193 | 0.217 | 0.239 |
| Wald chi-2 | 227.350 | 226.940 | 208.180 | 212.520 | 215.730 | 216.330 |
| Prob > chi-2 | 0.000 | 0.000 | 0.000 | 0.000 | 0.000 | 0.000 |
| AIC | 4001 | 4014 | 4053 | 4114 | 4192 | 4279 |
| BIC | 4107 | 4120 | 4159 | 4220 | 4298 | 4385 |
| y-hat-squared | 0.000 | 0.000 | 0.000 | 0.008 | 0.257 | 0.982 |
| [standard error] (p-value) |  |  |  |  |  |  |
