# Supplementary material for: Cost-effectiveness analysis of the diarrhea alleviation through zinc and oral rehydration therapy (DAZT) program in rural Gujarat India: an application of the net-benefit regression framework
Source: Cost Eff Resour Alloc. 2017 Jun 8;15:9. doi: 10.1186/s12962-017-0070-y (PMC5465559; doi:10.1186/s12962-017-0070-y)
Supplement: Supplementary file 4 — Additional file 4: Figure S1. Adjusted cost-effectiveness acceptability curves using the full set of covariates. [file 12962_2017_70_MOESM4_ESM.docx]

**Web Figure 1.** Adjusted cost-effectiveness acceptability curves using the full set of covariates
