# Supplementary material for: Cost-effectiveness analysis of the diarrhea alleviation through zinc and oral rehydration therapy (DAZT) program in rural Gujarat India: an application of the net-benefit regression framework
Source: Cost Eff Resour Alloc. 2017 Jun 8;15:9. doi: 10.1186/s12962-017-0070-y (PMC5465559; doi:10.1186/s12962-017-0070-y)
Supplement: Supplementary file 5 — Additional file 5: Table S4. Net benefit of the DAZT program relative to (baseline) conditions existing before the program—multivariable regression with interaction terms with full set of covariates and with ORS and zinc coverage as the effectiveness measure. [file 12962_2017_70_MOESM5_ESM.docx]

**dWeb Figure 5.** Cost-effectiveness acceptability curves: Rheingans selection of variables [30]
