# Supplementary material for: Cost-effectiveness analysis of the diarrhea alleviation through zinc and oral rehydration therapy (DAZT) program in rural Gujarat India: an application of the net-benefit regression framework
Source: Cost Eff Resour Alloc. 2017 Jun 8;15:9. doi: 10.1186/s12962-017-0070-y (PMC5465559; doi:10.1186/s12962-017-0070-y)
Supplement: Supplementary file 10 — Additional file 10: Figure S6. Cost-effectiveness acceptability curves: Rheingans selection of variables adjusted with interaction terms. [file 12962_2017_70_MOESM10_ESM.docx]

**Web Figure 6.** Cost-effectiveness acceptability curves: Rheingans selection of variables adjusted with interaction terms
