# Supplementary material for: Cost-effectiveness analysis of the diarrhea alleviation through zinc and oral rehydration therapy (DAZT) program in rural Gujarat India: an application of the net-benefit regression framework
Source: Cost Eff Resour Alloc. 2017 Jun 8;15:9. doi: 10.1186/s12962-017-0070-y (PMC5465559; doi:10.1186/s12962-017-0070-y)
Supplement: Supplementary file 11 — Additional file 11: Table S5. Net benefit of the DAZT program relative to status quo—multivariable regression with interaction terms with Rheingans et al. [30] set of interaction terms: ORS and zinc coverage as effectiveness measure. [file 12962_2017_70_MOESM11_ESM.docx]

**Web Table 5.** Net benefit of the DAZT program relative to status quo - Multivariable regression with interaction terms with Rheingans et al (2012) set of interaction terms: ORS and zinc coverage as effectiveness measure

| **Variable** | **NB with  = $0** | | **NB with  = $20** | | **NB with  = $40** | **NB with  = $60** | **NB with  = $80** | **NB with  = $100** | |
| --- | --- | --- | --- | --- | --- | --- | --- | --- | --- |
| Constant term | | -0.01 [1.80]  (0.50) | | 0.01 [1.77]  (0.50) | 0.03 [1.77]  (0.49) | 0.05 [1.80]  (0.49) | 0.07 [1.92]  (0.49) | 0.09 [1.89]  (0.48) |  |
| Study phase | | 0.02 [1.83]  (0.50) | | 0.43 [2.45]  (0.43) | 0.84 [3.80]  (0.41) | 1.24 [5.34]  (0.41) | 1.65 [6.96]  (0.41) | 2.06 [8.79]  (0.41) |  |
| **Treatment-covariate interactions** | |  | |  |  |  |  |  |  |
| Wealth index - 2nd quintile | | 2.61 [1.88]  (0.08) | | 1.89 [2.39]  (0.21) | 1.17 [3.33]  (0.36) | 0.45 [4.96]  (0.46) | -0.27 [6.27]  (0.48) | -0.98 [7.52]  (0.45) |  |
| Wealth index - 3rd quintile | | 1.51 [1.37]  (0.14) | | 0.62 [2.05]  (0.38) | -0.26 [3.06]  (0.47) | -1.14 [4.63]  (0.40) | -2.03 [5.74]  (0.36) | -2.91 [7.07]  (0.34) |  |
| Wealth index - 4th quintile | | 0.93 [1.11]  (0.20) | | 2.99 [1.97]  (0.07) | 5.06 [3.38]  (0.07) | 7.12 [4.89]  (0.07) | 9.19 [6.20]  (0.07) | 11.25 [7.72]  (0.07) |  |
| Wealth index - 5th quintile | | 1.71 [2.47]  (0.24) | | 4.08 [3.10]  (0.09) | 6.45 [5.06]  (0.10) | 8.82 [6.65]  (0.09) | 11.18 [8.41]  (0.09) | 13.55 [10.74]  (0.10) |  |
| Female child | | -0.51 [1.04]  (0.31) | | -0.69 [1.51]  (0.33) | -0.87 [2.16]  (0.34) | -1.05 [3.01]  (0.36) | -1.23 [3.99]  (0.38) | -1.41 [4.70]  (0.38) |  |
| Mother with primary education | | -1.85 [1.00]  (0.03) | | -0.69 [1.28]  (0.29) | 0.46 [1.85]  (0.40) | 1.62 [2.60]  (0.27) | 2.78 [3.48]  (0.21) | 3.94 [4.26]  (0.18) |  |
| Mother with secondary education | | 1.17 [1.68]  (0.24) | | 1.67 [2.15]  (0.22) | 2.18 [3.27]  (0.25) | 2.68 [4.31]  (0.27) | 3.19 [5.40]  (0.28) | 3.69 [6.78]  (0.29) |  |
| Child age | | -0.04 [0.04]  (0.20) | | -0.06 [0.05]  (0.13) | -0.08 [0.07]  (0.14) | -0.10 [0.09]  (0.13) | -0.12 [0.12]  (0.15) | -0.15 [0.15]  (0.16) |  |
| Blood in the stool | | 0.71 [1.75]  (0.34) | | 3.09 [2.87]  (0.14) | 5.46 [4.62]  (0.12) | 7.83 [6.72]  (0.12) | 10.20 [9.22]  (0.13) | 12.57 [11.03]  (0.13) |  |
| Duration of diarrhea | | -0.15 [0.62]  (0.40) | | 0.35 [0.68]  (0.30) | 0.86 [0.87]  (0.16) | 1.36 [1.14]  (0.12) | 1.87 [1.41]  (0.09) | 2.37 [1.78]  (0.09) |  |
| **Covariates** | |  | |  |  |  |  |  |  |
| Wealth index - 2nd quintile | | -2.82 [1.90]  (0.07) | | -2.51 [1.89]  (0.09) | -2.19 [1.99]  (0.14) | -1.88 [2.15]  (0.19) | -1.56 [2.35]  (0.25) | -1.25 [2.44]  (0.30) |  |
| Wealth index - 3rd quintile | | -1.85 [1.09]  (0.05) | | -1.88 [1.08]  (0.04) | -1.92 [1.06]  (0.04) | -1.95 [1.07]  (0.03) | -1.99 [1.10]  (0.04) | -2.02 [1.07]  (0.03) |  |
| Wealth index - 4th quintile | | -1.34 [0.84]  (0.06) | | -1.33 [0.84]  (0.06) | -1.32 [0.89]  (0.07) | -1.32 [0.88]  (0.07) | -1.31 [0.87]  (0.07) | -1.30 [0.90]  (0.07) |  |
| Wealth index - 5th quintile | | -2.27 [2.37]  (0.17) | | -1.67 [2.30]  (0.23) | -1.07 [2.51]  (0.34) | -0.47 [2.73]  (0.43) | 0.13 [3.02]  (0.48) | 0.73 [3.41]  (0.41) |  |
| Female child | | 0.83 [0.88]  (0.17) | | 0.88 [0.89]  (0.16) | 0.92 [0.96]  (0.17) | 0.97 [1.07]  (0.18) | 1.01 [1.22]  (0.20) | 1.06 [1.32]  (0.21) |  |
| Mother with primary education | | 1.49 [0.82]  (0.03) | | 1.48 [0.80]  (0.03) | 1.48 [0.82]  (0.04) | 1.48 [0.95]  (0.06) | 1.47 [1.10]  (0.09) | 1.47 [1.14]  (0.10) |  |
| Mother with secondary education | | -1.19 [1.58]  (0.23) | | -1.59 [1.55]  (0.15) | -2.00 [1.80]  (0.13) | -2.40 [1.93]  (0.11) | -2.81 [2.21]  (0.10) | -3.21 [2.54]  (0.10) |  |
| Child age | | 0.06 [0.04]  (0.06) | | 0.05 [0.04]  (0.09) | 0.04 [0.04]  (0.14) | 0.03 [0.04]  (0.21) | 0.03 [0.04]  (0.27) | 0.02 [0.05]  (0.35) |  |
| Blood in the stool | | -2.27 [1.56]  (0.07) | | -2.24 [1.59]  (0.08) | -2.21 [1.52]  (0.07) | -2.18 [1.57]  (0.08) | -2.15 [1.50]  (0.08) | -2.12 [1.55]  (0.09) |  |
| Duration of diarrhea | | -0.88 [0.62]  (0.08) | | -0.90 [0.61]  (0.07) | -0.91 [0.60]  (0.07) | -0.92 [0.61]  (0.07) | -0.93 [0.63]  (0.07) | -0.94 [0.61]  (0.06) |  |
|  | |  | |  |  |  |  |  |  |
| R-squared | | 0.0483 | | 0.088 | 0.0795 | 0.0913 | 0.0975 | 0.101 |  |
| Wald chi-2 | | 79.9 | | 40.5 | 54.85 | 63.48 | 68.59 | 73.37 |  |
| Prob > chi-2 | | 0.000 | | 0.007 | 0.000 | 0.000 | 0.000 | 0.000 |  |
| AIC | | 4067 | | 4409 | 4910 | 5312 | 5627 | 5883 |  |
| BIC | | 4164 | | 4507 | 5007 | 5409 | 5724 | 5980 |  |
| y-hat-squared | | 0.019 | | 0.253 | 0.788 | 0.836 | 0.632 | 0.520 |  |
| [standard error] (p-value) | |  | |  |  |  |  |  |  |
